# Use of Thomas splint in salvaging free flaps of the lower limb in violent postoperative patients

**DOI:** 10.4103/0970-0358.59301

**Published:** 2009

**Authors:** K. G. Bhaskara, Subhash M. Kale

**Affiliations:** Department of Plastic and Microvascular Surgery, Medical Trust Hospital, M.G.Road, Kochi-16, India

Sir,

Postoperative care like immobilization, monitoring, local warmth, and limb elevation of patients is very important for the first few days after free flaps until the anastomoses becomes stable.[1]

We found that most of our chronic alcoholic patients, who underwent free flap surgery for posttraumatic soft tissue loss, became very irritable, violent, and uncontrollable. They lost their orientation and did not obey any verbal instructions. This could be most commonly attributed to alcohol withdrawl syndrome[2] or could be the effect of head injury or general anesthesia.

Postoperatively, these violent patients are restless and move their limbs in bed which can lead to microvascular flap failure.[3] Their limbs can not be tied because of the operative site and fear of compression of vascular supply. We have found that the innovative use of the Thomas splint to immobilize the limbs of such patients, gives good stability even when these patients continued to be violent and restless.

**Figure 1:**
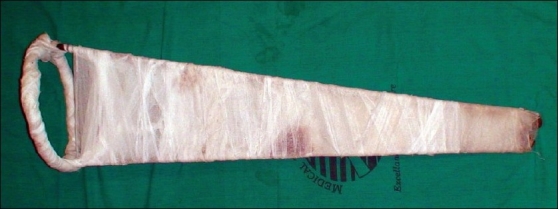
Thomas splint frame with bandage strapping

We use the Thomas splint on the operated limb to immobilize the knee joint[4] and secure the Thomas splint tight enough to the thigh and the knee as shown in Figure 1, so that patient can not move his leg. We keep the Thomas splint until the patient is co-operative and well oriented.

**Figure 2:**
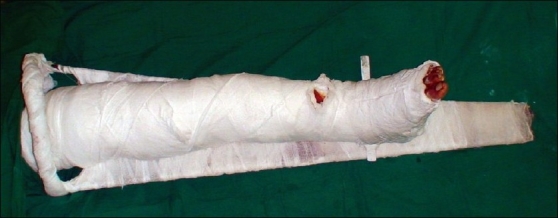
Patient's limb with free L.D. Muscle flap immobilized with Thomas splint with below knee de-rotation posterior POP slab

Figure 2 shows a below-knee posterior POP slab given for flap and fracture immobilization and to secure the skin graft in position with de-rotation and the use of a small metal plate. We can tie this plate tightly with the Thomas splint to immobilize leg. The below-knee slab is not enough to immobilize violent patient as they start to lift their leg, bend the knee, and flex their thigh. With the Thomas splint firmly in place, we were able to immobilize the entire lower limb without any direct tight pressure over the flap; and secure the slab with the splint.

Thus, we have salvaged our free flaps in more than ten violent patients with the Thomas splint. It is very difficult to control violent patients with sedatives or immobilizing them with above-knee POP slabs, since they usually break free. To restrict and decrease the patients' direct forces over the leg or the operated area we have used the Thomas splint firmly in place to immobilize the proximal two joints, the hip and the knee. We can strap or tie as many places to the Thomas splint frame on the side and tie it to the bed at the lower end, which is otherwise difficult with only a POP slab. It is a very cost-effective and easily available method with no disadvantages.
